# Cold housing environments: defining the problem for an appropriate policy response

**DOI:** 10.1057/s41271-023-00431-8

**Published:** 2023-07-29

**Authors:** Cynthia Faye Barlow, Lyrian Daniel, Rebecca Bentley, Emma Baker

**Affiliations:** 1 The Australian Centre for Housing Research, Faculty of Arts, Business, Law and Economics, University of Adelaide, Adelaide, SA 5005 Australia; 2 UniSA Creative, University of South Australia, Adelaide, SA 5000 Australia; 3 The Centre for Health Policy, Melbourne School of Population and Global Health, University of Melbourne, Melbourne, VIC 3010 Australia

**Keywords:** Building conditions, Cold temperature, Housing, Economic policy, Public health

## Abstract

Researchers across disciplines are increasing attention to cold housing environments. Public health, environmental and social sciences, architecture, and engineering each define and measure cold housing environments differently. Lack of standardisation hinders our ability to combine evidence, determine prevalence, understand who is most at risk––and to formulate policy responses. We conducted a systematic, cross-disciplinary review of literature to document the measures used. We examined benefits and limitations of each approach and propose a conceptualisation of cold housing: where temperature is too low to support optimal health and wellbeing of inhabitants, measured using one or a combination of economic, ‘objective’, or subjective approaches. More accurate data on home temperatures for all population groups, combined with an understanding of factors leading to cold homes, will enable appropriate policy response to reduce adverse health effects and costs. Policies targeting better building standards and energy subsidies both improve temperature conditions in housing environments.

**Supplementary Information:**

The online version contains supplementary material available at 10.1057/s41271-023-00431-8.

## Key messages

- Cold housing is a complex issue that spans a range of disciplines and methodological approaches
- Based on our systematic review, we define cold housing as: where temperature is too low to support optimal health and wellbeing of inhabitants, measured using one or a combination of economic, ‘objective’, or subjective approaches
- Policy intervention to alleviate cold housing therefore requires consideration of building design, health effects and cost of living pressures

## Introduction

Ideally, our housing environment provides us shelter and comfort. Because of design, materials, financial resources, or climate, the protection provided is highly variable. One increasingly important failing—as highlighted in recent work by the World Health Organization (WHO [1]—is the problem of cold. Cold home environments are a major and growing concern for governments [2–6], advocacy groups [7], and, in an era of rapidly rising energy costs, householders [8].

The topic of ‘cold housing environments’ challenges the research community as the ways disciplines define and measure this vary widely. The World Health Organization Housing and Health Guidelines define cold homes as those with an indoor temperature below 18 °C [1]. This followed a literature review of 11 articles focussed primarily on whether there were adverse health impacts from living in home environments less than 18 °C rather than in warmer homes [9]. The WHO conducted an additional literature review about specific health benefits from the presence of home insulation [10]. Seven of those 11 studies found living in an insulated home environments to be associated with improved health [10].

Others have considered cold home environments from the perspective of occupant satisfaction with conditions [11]. Identifying a suitable method for studying thermal comfort in people’s homes [12], financial ability to heat the home [13], perceived inability to improve temperature conditions [14], or difficulty heating the home due to building conditions [15]). There has also been a marked increase in literature regarding cold home environments in the past few years, following the WHO reviews (see Results section). Our focus differs from that of the WHO reviews as we focus on the manner in which researchers have measured ‘cold’ and defined ‘cold home environments’.

The variety of ‘cold homes’ definitions motivated us to explore if there might be a ‘correct’ way to define them.

- Is it best to limit the definition to a health viewpoint, with specific outcomes such as cardiovascular and respiratory health? Or, to consider also the mental health of inhabitants?
- Is the occupant’s perception of cold, or physical vulnerability to cold, more or less important than the actual temperature?
- Is it more important to define a standard of warmth versus cold, or to address the underlying conditions that cause homes to be cold in the first place, such as energy poverty, energy efficiency or building design and condition?

We systematically survey recent evidence on cold housing environments. The following section sets out our review methodology, followed by a critical overview of the different approaches to measuring cold housing environments, and discussion of emerging challenges. We conclude by proposing a working definition and measurement approach to align future work and support an appropriate policy response.

## Method

We searched titles, abstracts, and keywords on PubMed, Web of Science, Science Direct, Scopus, and Google Scholar, for publications from 2000 to 2022 (inclusive). The WHO reviews [9, 10] captured some topics in this literature up to 2018. Because our interest is broader, we focus on measurement or definition of cold, and work since the WHO reviews. We used search terms ‘measuring cold in homes’, ‘cold hous’, ‘indoor cold’, ‘cold hom’. Most studies we identified initially related to cold-climate countries. Because cold housing is also prevalent in warmer climates [16, 17], we included ‘temperature extremes in homes’, ‘heating homes in warm climates’, ‘heating challenges in temperate climate’, ‘heating in [country]’, for Spain, Portugal, and Greece, and we added ‘minimum home temperature’ and ‘heating gap’.

**Fig. 1:**
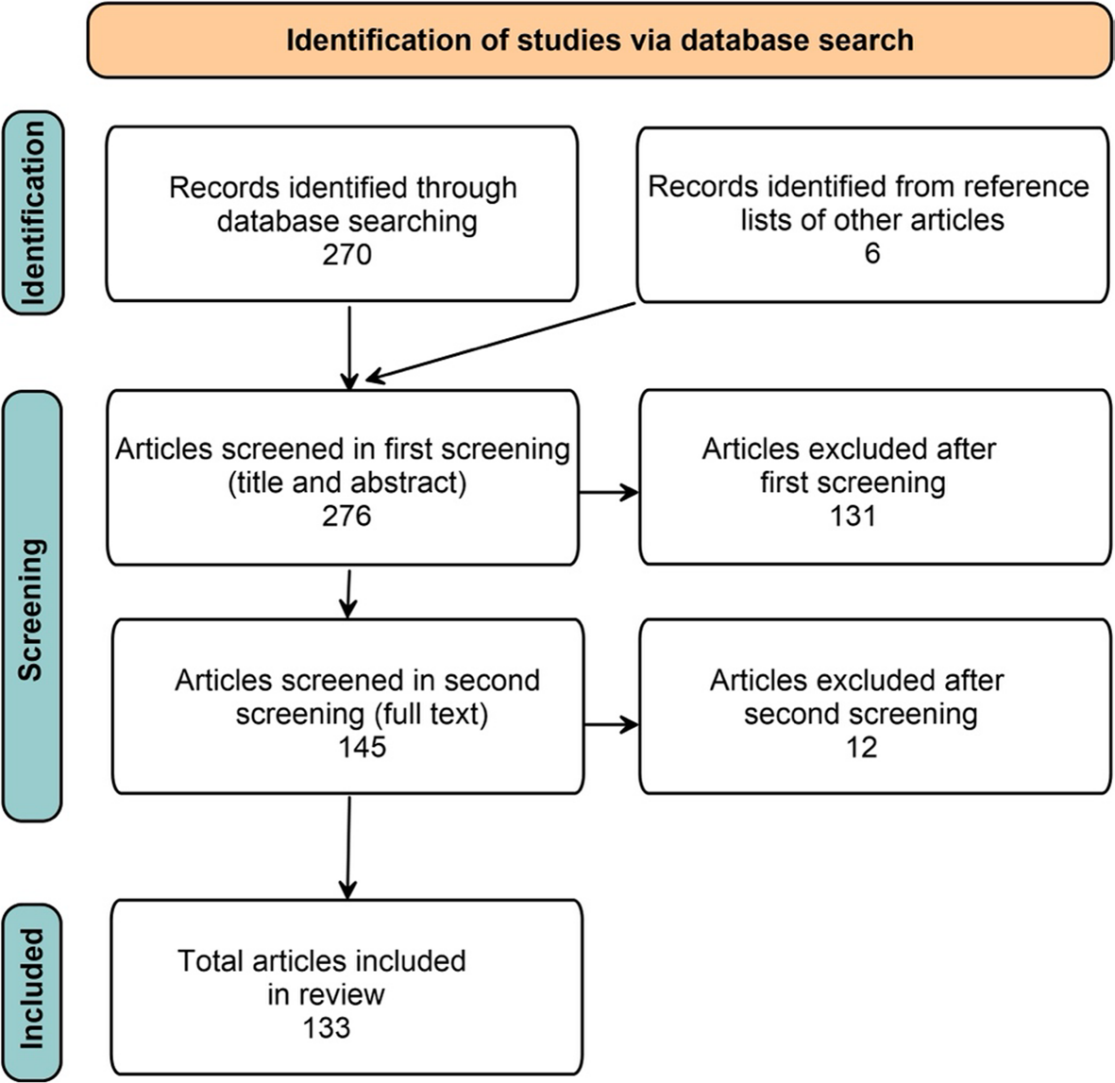
Summary of the literature review method

Based on titles and abstracts we identified a total of 270 publications (Fig. 1). We excluded 143 papers in our first or second screening because they did not provide a measure of coldness (62), studied animals not humans (26), focused on clinical research without consideration of home environments (43), or focused on emissions, fuels, building design or real estate sales (12). This produced 133 relevant publications for analysis. For each we assessed six features for thematic analysis:

1. Country where researchers conducted the study
2. Measures of cold
3. Factors used to assess cold housing
4. Methodology (equipment, questionnaire, meta-analysis of database)
5. Sample size (homes, participants)
6. Main findings of the study

## Results

The number of relevant articles has increased markedly––with almost half (62 of 133 papers) published in the last 5 years. In the 133 reviewed (see Supplementary Material for full list), the breadth of factors and diversity of measures of cold indicate that defining and addressing ‘cold home environments’ is complex.

We classified the methodological approaches for measuring cold housing environments in three categories: ‘objective’, ‘subjective’, and ‘economic’. Each provides a unique entry point for policy response (See Discussion). This categorisation follows the work of Foye [18] who examined the epistemological origins of housing research and approaches taken toward measuring housing outcomes. The next three subsections describe the range of measures and their specifications in the study of cold housing environments.

### Objective measures

Temperature is the primary objective measure of cold in homes, however, the method of measurement varied widely, as did sample numbers and duration of sampling. There is no consensus on what defines a cold home (Table 1). Health evidence points to 18 °C as a suitable minimum in general although higher temperatures may be needed where occupants are vulnerable to cold due to health or age [1]. Some studies recommended different temperatures for daytime and night-time [19], different lengths of time [20] or different rooms [21]. Reasons for the temperatures recommended differed among studies. Some relate to specific medical conditions or occupant comfort for population groups such as school children [2] or the elderly [22]. Occupant awareness of indoor temperatures can also improve health and well-being [23], leading to reduced use of medication [24].

**Table 1:** Home temperatures considered as a minimum or as cold

Literature	Minimum temperature advocated, or defining home as ‘cold’	Reason for recommendation
WHO [1]	18 °C	Protection of health
UK Parliament [5]	18 °C	Environmental housing standard
Mu et al. [25]	18.2 °C	Respiratory health
Shiue [26]	18 °C	Risk of elevated blood pressure
Public Health England (Wookey et al. [4])	18 °C daytime May be lower overnight	Protection of health
Hutchinson et al. [27]	16 °C	Identification of a home as ‘cold’
Critchley et al. [14]	18 °C living room 16 °C bedroom	Identification of a home as ‘cold’
Bouzarovski and Petrova [19]	21 °C day 17 °C night	Thermal comfort
Magalhães et al. [21]	21 °C living room 18 °C other occupied rooms	Adequate standard of warmth
Osman et al. [20]	21 °C for at least 9 h a day	Protection of health COPD patients
Tartarini et al. [22]	19.1 °C	Comfort of aged care residents
Reyes et al. [28]	21 °C	Identification of a home as ‘cold’
CIBSE [29]	22–23 °C living room	Comfort
Simoes et al. [30]	20 °C	Portuguese building regulations
US EPA [2]	19.7 °C	School regulations
ASHRAE [3]	17.5 °C	Comfort
Shiue [26]	16 °C	Elevated blood pressure risk

Of the 64 studies using temperature sensors, many listed the make and model of sensor (see Supplementary Material). Others simply described the sensors, such as ‘portable’ [31], or ‘electronic’ [32]. For studies with monitored temperature, median participant numbers included 112 homes, monitored over 2.5 weeks. Umishio et al. [33] monitored 2190 Japanese homes for 2 weeks each, Yu et al. [34] collected measurements in 527 Chinese homes, but only briefly (< 1 h), as they conducted an interview. Pullinger et al. [35] measured temperature in a relatively large number of homes in the United Kingdom (UK) for a lengthy period (255 homes between 55 and 673 days each). Fan et al. [36] measured temperature in only 10 Chinese homes for 1 week.

### Subjective measures

It is important to consider the factors that influence cold home environments, even though they are not, of themselves, measures of coldness. These include occupant perception of comfort, energy usage, economic factors (energy poverty), building conditions and behavioural aspects. Such underlying aspects are crucial for understanding ways to improve temperature conditions in homes.

We summarise measures used to describe cold home environments in Table 2. The dominant subjective measure is an occupant's perception of thermal comfort. The American Society of Heating Refrigerating and Air Conditioning: Engineers Thermal provide a commonly used definition of thermal comfort: the “condition of mind that expresses satisfaction with the thermal environment and is assessed by subjective evaluation” [3]. Some define thermal comfort as a mental state where occupants are not distracted by the coldness of the environment [11]. In the absence of physical temperature measurements in homes, one Australian study used these subjective measures to inform health monitoring [37].

**Table 2:** Measures of cold employed in reviewed literature

Measure of cold	Number of studies	Details
Indoor air temperature	64	• Sensor or logger, listed in Supplementary Appendix A
Occupant perceived thermal comfort	45	• Surveys (*n* = 15) [46] • Interviews (*n* = 11) [11] o telephone [47] o e-interviews [48] and o group interviews [49] • PMV (or ASHRAE) scale (8) [50] • Likert-scale questions (3) [51] • McIntyre scale (1) [52] • Safe and healthy temperatures [53] • Clothing arrangement, recent physical activity and operation of windows [52]
Energy use or cost	23	• Actual use statistics [54] • Heating degree days [55] • Thermostat settings [56] • Heating hours [39] • Presence of central or other heating [57]
Energy poverty	42	• Inability to keep the home warm [13] • Inability to pay energy bills on time (8) [44] • Gini index (income after tax and welfare) [55, 58] • Simple index (required domestic fuel costs/income > 10%) [59] • More complex equations [60] • Thermal adaptive behaviours [22] • Energy cost (22 studies e.g. [27]) • Fuel poverty [61]
Secondary analyses	12	• Previously collected data [62] o in-home sensor studies [63] • Survey data [64] • Eurostat 2019 [50, 55, 58] • French database of the healthcare and insurance survey [65] • National surveys (Japan [66], New Zealand [67]) • Hospital episode and population statistics [66, 68]
Heating systems and behaviours	19	E.g. Wright [69]
Insulation or thermal efficiency	10	E.g. Hamilton, Davies, Ridley, Oreszczyn, Barrett, Lowe, Hong, Wilkinson and Chalabi [15]
Damp/mould	10	E.g. Butler, Williams, Tukuitonga and Paterson [70]
Thermostat settings	6	E.g. Shipworth [56]
Other measures	12	E.g. Heating degree days [71]

Examples of literature given are for each point in this table but are not exhaustive; see Supplementary Material for further detail. Also note that some studies use multiple measures of cold

Researchers measured thermal comfort using various scales, such as predicted mean vote (PMV) [38] and the Likert scale [39] and techniques such as interviews and surveys (Table 2). Use of surveys rather than physical measurement in homes allowed for larger sample numbers, the median number of participants being 342, or 276 excluding secondary data analyses. Survey-based studies ranged from four Spanish households, based on an interview and intervention study [40]; to 193,492 data points based on surveys of household, income and labour dynamics in Australia (HILDA) [41].

Perception of thermal comfort depends on physical factors including air velocity, mean radiant temperature, and stratification [3, 5]. First, air velocity of 1 ms^−1^ causes air to feel 1 °C colder [42]. Second, if the wall temperature of a house is less than that of the air in the room, heat will radiate from a person within that room towards the wall, causing them to feel cold [42]. Third, hot air rises, meaning that air temperature within a room is stratified, people within a room may therefore have cold feet, causing discomfort [5]. Aside from these physical factors, individual perception of and adaptation to cold may vary [43]. Those preferring lower temperatures reported less anxiety and depression from living in a ‘cold’ home than those with limited control over their home environment [14]. Provision of heating does not necessarily lead to improved perception of thermal comfort [44], particularly where occupants feel constrained by energy cost, as detailed below. The perception of being cold in the home environment, over the long term, negatively impacts the occupant's mental health [45]. Hence, we cannot simply consider temperature and ignore occupant perception.

### Economic measures

Studies reviewed show that cold home environments and energy-poverty (Table 2) are positively related [72, 73]. Nakajima, Schmidt, Fänge, Ono and Ikaga [74] found that despite the temperature of the home, perceived health impacts were more likely when coupled with energy poverty. Naicker et al. [16] explain this by the limited availability of coping strategies in lower socio-economic communities, where low-cost housing is poorly constructed. Energy use or cost provides a measure of how much a home is heated, but it does not indicate fully the comfort level or actual temperature of the home [56]. Energy usage in English homes has increased, despite no change in thermostat settings over the same period, potentially because larger areas are now being heated, or for longer time periods [56]. During COVID lockdowns, energy consumption by United States (U.S.) households increased up to 30%, due to higher occupancy patterns and need for increased use of heating and air conditioning [75]. Similarly, energy usage does not capture fully a household’s ability to heat their home as some homes may be more, or less heat efficient [39, 68, 76]. Thus, for policy, it may be appropriate to target reducing fuel poverty for the most vulnerable categories of individuals, for example, chronic patients who experience difficulty heating their homes [65].

## Discussion

Researchers commonly situated investigation of cold housing environments in relation to factors potentially driving exposure, or specific vulnerable cohorts. Therefore, there must be some way to benchmark a ‘cold housing environment’; measurement of temperature is the most logical way to objectively compare one home to another. It is also important to consider the occupants of each home, whose environmental needs vary due to medical conditions, age, or use of the home. To address the problem of cold home environments effectively we must look beyond temperature to the factors that cause homes to be cold. Thus, next we discuss economic and material drivers of cold housing environments, as well as issues of occupancy and vulnerability. For shaping policy, temperature in homes is very difficult to address directly. Why? Researchers seldom measure temperature in homes and government authorities cannot easily impose controls on temperature in individual households. Instead, we must provide households with capacity to maintain their indoor environment at a healthy temperature. Policy avenues to achieve this include building codes and economic policy, such as energy subsidies.

### Building conditions

Building conditions do not define the temperature of a home, but they play an important role in determining which home environments are likely to be ‘cold’. Homes with poor building conditions, including a lack of insulation or heating [33], damp [32] and mould [67], were more likely those in which occupants reported feeling cold or in which temperatures were below WHO recommendations.

Energy efficiency is an important factor for maintaining warmth and wellbeing in homes. In the U.S., the California Energy Commission [77] updates building energy efficiency standards every 3 years. Others operate similarly, including the European Commission [78] Energy Performance of Buildings Directive and Australian National Construction Code [6, 79]. Making homes more energy efficient also reduces energy demand and greenhouse gas emissions.

Policy governing energy efficiency of new housing is, however, of little benefit to those living in housing of poor quality. Thus, policy makers need to focus on retrofitting of older housing stock [80]. In the U.S., a trial involving 53 homes found that retrofitting improved energy efficiency, particularly in winter [80]. In New Zealand, a successful community trial of retrofitting 1350 homes led to policy change to sponsor retrofit of insulation and efficient heating into existing homes [81]. Similarly, Australian building policy aims to retrofit older housing stock from 2025 onwards [6]. Unless specifically targeted, retrofitting policies may fail to benefit those who are most vulnerable; ‘universal’ policies may actually increase inequality, as groups suffering most from fuel poverty are least likely to participate [82]. Thus, better policies ensure that retrofits are practically and economically available to the most vulnerable members of the community.

Policy must also address rental tenants, who lack capacity to change building conditions. Government policy often fails to motivate private sector landlords to improve energy efficiency [83]. In the UK, policy to improve social housing conditions has met with mixed success [84], improving conditions, but also limiting availability of housing for the most vulnerable. Effective intervention requires targeted incentives [83], or introduction of minimum standards such as energy efficiency [85], provision of heating and ventilation [86], or for installing insulation.

The impact of energy efficiency varies. Casquero-Modrego and Goñi-Modrego [40] found that retrofit of Spanish homes did not significantly reduce energy consumption but did lead to the perception of greater thermal comfort. Elsewhere, results have been more positive. In Wales [87], energy-efficiency investment in the UK increased subjective wellbeing and the researchers found links to psychosocial intermediaries (increased thermal comfort, reduced reports of having to live with cold, fewer financial difficulties, and reduced social isolation) conducive to better health. Energy efficiency interventions also had differentiated effects on cold-related mortality in men and women, with effects also varying by cause, educational level, and age [88]. The WHO recommends installation of insulation in new housing and retrofit in existing housing [1], for improved health [10].

Mould and damp are closely related to cold in homes and some studies used them as an indicator (*n* = 10, see Supplementary Material and Cotter, Monahan, McAvoy and Goodman [89]). This amounts to a recognition that damp and mould are often synonymous with cold housing [32, 90]. Respiration of mould can lead to health problems, such as fungal respiratory infections such as Aspergillosis, Histoplasmosis and Coccidioidomycosis [79]. Damp and mouldy housing accounts for a substantial proportion of the burden of disease, as shown in the U.S. [91] and New Zealand [92]. Improving the thermal quality of housing to eliminate damp and mould and producing a comfortable temperature through the house had a positive impact on the health of the residents [68], and substantially reduced total hospitalisation costs and potentially improved quality of life [92]. Thus, thermal quality produced financial benefits for occupants and indirectly for government health providers [68].

Evidence is emerging, however, that in warmer-climate countries, such as Australia [79, 93] and Spain [94], increased energy-efficiency through changed national construction code rules has increased the occurrence of wintertime condensation and mould growth. Even in cooler countries, such as Greenland [95], researchers noted that keeping homes warm often compromises ventilation. Condensation of moisture typically occurs on cold surfaces, such as window frames and uninsulated ceilings [79]. Ventilating roof spaces may even exacerbate condensation, by increasing the temperature differential [79]. This has led to a higher proportion of mould-damaged buildings, for example, 50% in Australia compared to that of Europe (45%), U.S. (40%) and Canada (30%) [96]. Building material choices and improved airtightness also contribute to condensation [79, 93, 96]. Solutions developed in colder climates, that focus on airtightness, may not be applicable to warmer climates. Although evidence that inability to keep the home warm enough in winter is more strongly associated with adverse health outcomes than is damp housing [97], building policies targeting warmth must not to compromise necessary ventilation.

### Occupancy and vulnerable cohorts

Several of the studies (*n* = 28, Supplementary Material), including Rudge and Gilchrist [68], focussed on the aged population, people more susceptible to health consequences from cold housing [98, 99]. Older people are particularly at risk from energy inefficient, cold homes [66, 99, 100]. This has led the UK to implement public-health-driven energy efficiency housing policy interventions [68]. A more comprehensive understanding of who was at risk from cold homes would enable better health protection for the aged community [101]. Younger people are also vulnerable to cold homes if, for economic reasons, they are unable to keep their houses warm [48, 102].

The UK government has addressed this need by offering energy discounts to pensioners and those on low incomes [103]. Similar schemes are available in the U.S. [104] and Australia [105]. With rising energy prices, subsidies alone may be insufficient to enable the most vulnerable to effectively heat their homes [106], particularly where poor building conditions are a factor. Support for these vulnerable community members will require a combination of energy subsidies with improvement of housing conditions to maintain safe indoor winter temperatures [106].

## Conclusion

A definition of a cold home environment that captures the complexity seen in the research literature would include physical temperature, occupant perception, and the practical and economic capacity to heat the home. Physical temperature thresholds enable direct comparison of homes and indicate likely health impacts from cold homes. Individuals’ needs vary such that no one temperature defines a ‘warm’ or ‘healthy’ home environment. Cold home environments require policy-makers to consider factors that influence them, economic factors (energy poverty) and building conditions (including insulation and heating). Hence, we define a cold home as one where the temperature is too low to support optimal health and wellbeing of inhabitants. Measurement of cold home environments should include data on temperature across the population; occupant age and health; economic capacity to heat the home and on inhabitant’s perception of thermal comfort.

## Supplementary Information

Below is the link to the electronic supplementary material.

Supplementary file1 (PDF 195 kb)

## Data Availability

All data used for this paper are provided in the supplementary material.

## Abbreviations

HILDA: Household, income and labour dynamics in Australia survey
PMV: Predicted mean vote
WHO: World Health Organization
