# Supplementary material for: Cold housing environments: defining the problem for an appropriate policy response
Source: J Public Health Policy. 2023 Jul 29;44(3):370–85. doi: 10.1057/s41271-023-00431-8 (PMC10484804; doi:10.1057/s41271-023-00431-8)
Supplement: Supplementary file 1 — Supplementary file1 (PDF 195 kb) [file 41271_2023_431_MOESM1_ESM.pdf]

Appendix A: Literature cited

Literature cited	Country	Measures of cold	Factors considered in assessment of cold housing	Methodology employed			Sample size				Main findings of the study	Subject
				Measurement method	Detail of method	Sensor type	number of participants	number of surveys	number of homes	duration		
Ahmed, A. I., R. S. McLeod and M. Gustin (2021). Forecasting underheating in dwellings to detect excess winter mortality risks using time series models. Applied Energy 286.	UK	Indoor temperature, gas consumption.	housing conditions, air temperature	Sensors, gas meters.	Quantitative, mathematical modelling.	Temperature, gas meter.			20		Predicting indoor temperatures for modelling health outcomes	Public Health
Ahrentzen, S., J. Erickson and E. Fonseca (2016). Thermal and health outcomes of energy efficiency retrofits of homes of older adults. Indoor air 26(4): 582-593.	USA	Temperature, relative humidity, and air infiltration. Perception of temperature quality.	housung conditions, occupant comfort (subjective measure), air temperature			HOBO data loggers U10-001 and U10-003	57	57	53	5 days (before and after)	Retrofit improves energy efficiency, more about heat than cold.	Thermal comfort
Ambrose, M., et al. The evaluation of the 5-star energy efficiency standard for residential buildings. Commonwealth of Australia, Canberra (2013).	Australia (Melbourne, Brisbane, Adelaide)	Temperature, electricity use.	temperature, housing conditions	Sensor.		Thermochron button cell data loggers			438	>1 year	Energy efficiency, didn't mention how cold homes were.	Miscellaneous
ASHRAE (2004). Thermal environmental conditions for human occupancy. ISSN 1041-2336	USA	Temperature, influence of humidity, air movement etc.	temperature, humidity, additional factors	Discussion only							Physical factors affecting perception of cold.	Thermal comfort
Andersen, R. V., J. Toftum, K. K. Andersen and B. W. Olesen (2009). Survey of occupant behaviour and control of indoor environment in Danish dwellings. Energy and Buildings 41(1): 11-16.	EU Denmark	Thermal sensation votes.	housung conditions, occupant comfort (subjective measure), air temperature	Survey, targeted, by mail then email (winter).	Statitsical analysis, does a particular feature (eg. Outdoor temp, window open) affect energy use behaviour.		636 (winter), 933 summer	636			Calculation of energy consumption.	Thermal comfort
Barrella R, Linares JI, Romero JC, Arenas E, Centeno E. Does cash money solve energy poverty? Assessing the impact of household heating allowances in Spain. Energy Research & Social Science. 2021 Oct 1;80:102216.	EU Spain	Energy expenditure	socioeconomic status, housing condition, occupant health, air temperature								Subsidising energy bills may not be as effective as also tackling poor housing conditions.	Energy Poverty
BEN H, SUNIKKA-BLANK M. A socio-technical approach to thermal comfort and heating behaviour in UK homes. In Proceedings of International Conference CISBAT 2015 Future Buildings and Districts Sustainability from Nano to Urban Scale 2015 (No. CONF, pp. 339-344). LESO-PB, EPFL.	UK	Thermal comfort, heating behaviour.	occupant comfort (subjective measure), occupancy patterns	Data loggers (temperature? Doesn't specify).	Observations, photo records, diary records, data logger monitoring, questionnaire, interviews.				14		Significant gap between heating behaviour and thermal comfort, and that the provision of heating does not necessarily lead to high level of thermal comfort satisfaction. Comfort seems to be a mental state where you would not be distracted from the adversity of environment, unawareness of the surroundings. Others defined comfort as being reasonably warm, feeling relaxed.	Thermal comfort
Boemi, S. N. and A. M. Papadopoulos (2019). Energy poverty and energy efficiency improvements: A longitudinal approach of the Hellenic households. Energy and Buildings 197: 242-250.	EU Greece	Heating hours, mould, energy cost, thermal comfort.	climate (HDD), economic factors, housing conditions, occupant comfort (subjective measure)	Questionnaire, meter readings (gas, electricity, oil), interview.	Quantitative, longitudinal analysis, statistical observations, likert scale.		491	491			Impact of improving energy efficiency on energy poverty.	Energy Poverty
Boomsma, C., J. Goodhew, S. Goodhew and S. Pahl (2016). Improving the visibility of energy use in home heating in England: Thermal images and the role of visual tailoring. Energy Research & Social Science 14: 111-121.	UK	Thermal imaging.	housing conditions		Intervention, control group, post-intervention survey.		980				Thermal imaging leads to energy efficiency.	Miscellaneous
Bouzarovski, S. (2014). Energy poverty in the European Union: Landscapes of vulnerability. Wiley Interdisciplinary Reviews: Energy and Environment 3(3): 276-289.	EU	Arrears on energy bills, inadequate insulation, disproportionately high energy expenditure, unable to keep home warm.	economic status, occupant comfort (subjective measurement), housing condition,	Gleaned from published SILC data, Eurostat's Statistics on Income and Living Conditions (SILC) survey.	Secondary analysis of Eurostat's Statistics on Income and Living Conditions (SILC) survey.		16,000 Polish alone	16,000			Factors influencing energy poverty	Energy Poverty
Burholt, V. and G. Windle (2006). Keeping warm? Self-reported housing and home energy efficiency factors impacting on older people heating homes in North Wales. Energy Policy 34(10): 1198-1208.	UK		economic factors, housing conditions, occupant comfort (subjective measure)	Questionnaire.	Quantitative, statistical analyis, likert scale.		421				Housing condition, lack of energy efficiency and socio economic factors make heating more difficult.	Energy Poverty

Butler, D. and G. Sherriff (2017). 'It's normal to have damp': Using a qualitative psychological approach to analyse the lived experience of energy vulnerability among young adult households. Indoor and Built Environment 26(7): 964-979.	UK		housing condition, economic status, occupant age (youth)	Interviews.	Interpretative phenomenological analysis (IPS).	NA	6				Energy poverty of young-adult households.	Energy Poverty
Butler, S., M. Williams, C. Tukuitonga and J. Paterson (2003). Problems with damp and cold housing among Pacific families in New Zealand. New Zealand Medical Journal 116(1177).	New Zealand	Dampness, mould, cold.	air temperature, housung conditions, occupant health	interviews,	Quantitative, edinburgh postnatal dweperssionn scale, Statistical. Univariate and multivariate logistic regression procedure.		1376				Damp, cold housing relation to maternal deperssion and asthma.	Public Health
Camprubí, L., D. Malmusi, R. Mehdipanah, L. Palència, A. Molnar, C. Muntaner and C. Borrell (2016). Façade insulation retrofitting policy implementation process and its effects on health equity determinants: A realist review. Energy Policy 91: 304-314.	Global (review)		occupant health, housing condition, economic status	Review of energy poverty papers.	Understanding why different social groups vary in uptake of interventions for energy efficiency.						Insulation improves health benefits for most disadvantaged groups, universal policies are more likely to increase inequality	Energy Poverty
Carrere, J., A. Peralta, L. Oliveras, M. J. López, M. Marí-Dell'Olmo, J. Benach and A. M. Novoa (2022). Spain Gaceta Sanitaria 35: 438-444.	EU Spain	Inability to keep the home warm or inability to pay utility bills on time or presence of dampness and rot in the dwelling.	occupant health, housing condition, economic status	Secondary analysis of public health survey. Barcelona Public Health Survey (2016).	Association between health status and energy poverty intensity was estimated with multivariate models.		1500	1,500			Public health policy is needed to tackle energy poverty in Spain	Energy Poverty
Casquero-Modrego, N. and M. Goñi-Modrego (2019). Energy retrofit of an existing affordable building envelope in Spain, case study. Sustainable Cities and Society 44: 395-405.	EU Spain	Temperature, humidity, theremal energy of heating system, perception of thermal comfort.	air temperature, occupant comfort (subjective measure), housing conditions	Sensors, interview.	Quantitative, experimental (base and control group) multiple sensors in each home.	Temperature (HOBO), thermal meter (multical).		4	4		Retrofit didnt significantly reduce energy consumption , but did improve perception of thermal comfort.	Thermal comfort
Castaño-Rosa, R., J. Solís-Guzmán and M. Marrero (2020). A novel Index of Vulnerable Homes: Findings from application in Spain. Indoor and Built Environment 29(3): 311-330.	EU Spain	Monetary cost, energy and thermal comfort.	occupant health, housing condition, economic status, occupant comfort (subjective measurement), occupancy patterns	Secondary analysis, modelling. Considered occupancy rate of rooms (number of people) and hours of occupancy.	Multi-dimensional index is defined which relates technical aspects (characteristics of the dwelling) and social aspects (quality of life of households). European Quality of Life-5 Dimensions' (EQ-5D)79–81 questionnaire.						Multi-dimensional index is defined which relates technical aspects (characteristics of the dwelling) and social aspects (quality of life of households).	Energy Poverty
Charlier, D., B. Legendre and O. Ricci (2021). Measuring fuel poverty in tropical territories: A latent class model. World Development 140.	EU Guadeloupe, Martinique, Réunion and Guiana (French territories)	Availability hot water, insulation, electricity, cooking fuel type, air conditioning.	housing condition, economic status	Secondary analysis 2013 French housing survey (INSEE, 2013).	Latent class model (LCM) methodology to accurately assess fuel poverty in tropical areas using observable objective characteristics of decent, safe and healthy dwellings.						Identify a target group of households that should be a top priority in fighting fuel poverty in tropical regions.	Energy Poverty
CIBSE. CIBSE Concise Handbook - 3rd Ed., CIBSE ISBN: 9781903287941; https://cis.ihs.com/CIS/document/287866 2008. p. 3.	UK	Air temperature.	housing conditions, air temperature								Building codes. Recommends winter living room operative temperatures in the range of 22–23 °C.	Thermal Comfort
Conceição, E. Z. E., M. M. J. R. Lúcio and M. C. Lopes (2008). Application of an indoor greenhouse in the energy and thermal comfort performance in a kindergarten school building in the South of Portugal in winter conditions. WSEAS Transactions on Environment and Development 4(8): 644-654.	EU Portugal	Temperature, comfort level (simulation).	housung conditions, air temperature		Mass balancde simulation model.				1		Thermal building response.	Thermal comfort
Cotter, N., E. Monahan, H. McAvoy and P. Goodman (2012). Coping with the cold - Exploring relationships between cold housing, health and social wellbeing in a sample of older people in Ireland. Quality in Ageing and Older Adults 13(1): 38-47.	EU Ireland	Heating presence and cost, perception of damp/draught.	health of occupants, age of occupants, socioecomimic status, occupant comfort (subjective measure)		Mixed method, quantitative and behavioural, statistical.	NA	722				Cold homes associated with difficulty paying for heating, ill health and social exclusion.	Energy Poverty
Critchley, R., J. Gilbertson, M. Grimsley and G. Green (2007). Living in cold homes after heating improvements: Evidence from Warm-Front, England's Home Energy Efficiency Scheme. Applied Energy 84(2): 147-158.	UK	Air temperature, perception.	air temperature, housung conditions, occupant comfort (subjective measure	temp strip (occupants wriote down twice daily), interview	Qunatitative, statistical (binary logic regression), before/after 'warm front'.		888 temp, 79 interview	79	888		Older homes have unsatisfactory heating,m even aftyer funded improvements 'warm front'.	Public Health

Critchley, R., J. Gilbertson, M. Grimsley, G. Green and W. F. S. Group (2007). Living in cold homes after heating improvements: evidence from Warm-Front, England's Home Energy Efficiency Scheme. Applied Energy 84(2): 147-158.	UK	Temperature diary.	air temperature, housung conditions, occupant comfort (subjective measure)	also 79 interviews to understand data	Telephone interview, those preferring lower temperatures for health or other reasons, report less anxiety and depression than those with limited control over their home environment. cold homes, defined as those where either mean bedroom temperature over the measuring period fell	Temperature strip, manually recorded.			888	1-2 weeks before and after	Did 'warm front' interventions work?	Public Health
Croxford, B. (2009). The effect of cold homes on health: Evidence from the LARES study. Housing and Health in Europe: The WHO LARES Project: 142-154.	EU	Perception of temperature, heating, mould, condensation, draughtiness/air tightness, insulation.	occupant comfort (subjective measure), housing conditions, occupant health	LARES study (Large Analysis and Review of European housing and health Status).	Statistical association between health ooutcomes and paramaters measured.		8519	8,519	3374		Statistical association between health ooutcomes and paramaters measured.	Thermal comfort
Daniel, L., E. Baker and T. Williamson (2018). Residential wintertime comfort in a temperate Australian climate. Proceedings of 10th Windsor Conference: Rethinking Comfort.	Australia (Adelaide)	Temperature sensors.	housung conditions, air temperature, occupant comfort (subjective measure)						19	4 months	People in cold homes are not statisfied with thermal comf level.	Thermal comfort
Daniel, L., E. Baker and T. Williamson (2019). Cold housing in mild-climate countries: A study of indoor environmental quality and comfort preferences in homes, Adelaide, Australia. Building and Environment 151: 207-218.	Australia (Adelaide)	Indoor temperature, self- reported heatin and cooling practices and attitudes, energy usage, thermal comfort perception.	air temperature, occupant comfort (subjective measure), housing conditions, occupancy patterns	HOBO temperature loggers,	Paper survey, interviews, Responded on ASHRAE sensation scale, the three-point McIntyre preference scale, descriptors of their clothing arrangement, recent physical activity and operation of windows, fans, heating and cooling as well as qualitative comments.						Cold housing is a real, immediate problem in Australian households.	Thermal comfort
Daniel, L., E. Baker, A. Beer and N. T. A. Pham (2021). Cold housing: evidence, risk and vulnerability. Housing Studies 36(1): 110-130.	Australia (Adelaide)	Ability to heat home, energy expenditure.	age of occupants, socioecomimic status, occupant comfort (subjective measure)		Interviews (HILDA survey), used 15 waves between 2001 and 2016, mixed methods (quant and quant), critical evaluation of two theoiretical develipments, descriptive analysis of HILDA stats.		193,494	193,494			Tenure, employment, age & income affect ability to keep warm.	Energy Poverty
De Chavez, A. C., J. Gilbertson, A. M. Tod, P. Nelson, V. Powell-Hoyland, C. Homer, A. Lusambili and B. Thomas (2017). Using environmental monitoring to complement in-depth qualitative interviews in cold homes research. Indoor and Built Environment 26(7): 937-950.	UK	Temperature, self-reported behaviours.	temperature, economic factios, occupant health, occupant comfort (subjective measure)	Secondary analysis of Tiny Tag2, interviews, participant diary, self-reported heating analysis.	Based on Review of 6 Mixed-methods research studies.	TinyTag2	3489…six studies with varying n's				Meta analysis of the interface between qualitative and quanttative studies.	Miscellaneous
Environmental housing standards. Post note 650 September 2021 UK Parlaiment 2021 https://researchbriefings.files.parliament.uk/documents/POST-PN-0650/POST-PN-0650.pdf	UK	Housing standards.	air temperature, housung conditions,	**Environmental housing standards. Post note 650 September 2021**								Public Health
Eon C, Morrison GM, Byrne J. Unraveling everyday heating practices in residential homes. Energy Procedia. 2017 Sep 1;121:198-205	Au	Heating behaviour, electricity usage	housing condition, occupant comfort (subjective measure), air temperature, occupancy patterns						10	1 year	Energy usage and heating behaviours.	Energy Poverty
Erickson, V. L. and A. E. Cerpa (2012). Thermovote: participatory sensing for efficient building hvac conditioning. Proceedings of the Fourth ACM Workshop on Embedded Sensing Systems for Energy-Efficiency in Buildings.	USA	Thermovoting on thermal comfort to change air conditioning controls.	housung conditions, air temperature, occupant comfort (subjective measure)		Compared control with thermovoting compared to predicting conmfort based on outside tempergature etc.		39	39			Setting temperature for HVAC systems.	Thermal comfort
Evans, J., S. Hyndman, S. Stewart-Brown, D. Smith and S. Petersen (2000). An epidemiological study of the relative importance of damp housing in relation to adult health. Journal of Epidemiology and Community Health 54(9): 677-686.	UK	Dampness.	housing conditions, occupant health		Questionnaire (postal), to reandom sample of adults. 64% response rate.		8889				Cold more than damp homes are associated with health outcomes.	Public Health
Fan, G., J. Xie, H. Yoshino, U. Yanagi, K. Hasegawa, N. Kagi, T. Goto, Q. Zhang, C. Wang and J. Liu (2018). Indoor environmental conditions in urban and rural homes with older people during heating season: A case in cold region, China. Energy and Buildings 167: 334-346.	China	Temperature, relative humidity, fungi.	air temperature, housing conditions, air quality, occupant health	Sensors, visits.	T-test, pearson's correlation to see difference between urban and rural.	A three-channel data logging instrument (TR-76Ui, T&D Co., Japan;			10	1 week	Urban warmer than rural homes.	Public Health
Forcada, N., M. Gangolells, M. Casals, B. Tejedor, M. Macarulla and K. Gaspar (2021). Field study on thermal comfort in nursing homes in heated environments. Energy and Buildings 244: 111032.	EU Spain	Air temperature, relative humidity, air velocity, Radiant temperature, questionnaire on thermal comfort.	housung conditions, air temperature, occupant comfort (subjective measure), occupant age			Delta Ohm HD32.1			5	3 months	Residents were less sensitive to variations in temp than caregivers were, partly because of clothing adjustments.	Thermal comfort

Galvin, R. (2019). Letting the Gini out of the fuel poverty bottle? Correlating cold homes and income inequality in European Union countries. Energy Research and Social Science 58.	EU	Gini index, GDP/capita and heating degree days.	economic status, occupant comfort (subjective measurement), climate (heating degree days)	Literature review.	Gini index as a measure of income inequality; GDP per capita; the number of heating degree days; and several other likely determinants of energy poverty. Used Eurostat statistics (Eurostat, 2019). They cover all 28 EU countries in all years 2009–2017.						Income inequality is linked to inability to keep warm at home.	Energy Poverty
Galvin, R. (2019). Inequality and Energy: How Extremes of Wealth and Poverty in High Income Countries Affect CO2 Emissions and Access to Energy. EU: 145-171.	EU	Gini index.	economic status, climate (heating degree days), housing condition	Literature review.	Gini index as a measure of income inequality; GDP per capita; the number of heating degree days; and several other likely determinants of energy poverty. Used Eurostat statistics (Eurostat, 2019). They cover all 28 EU countries in all years 2009–2017.						Gini coefficient as measure of income after tax/welfare, is predictor of cold homes.	Energy Poverty
Ganem, C., H. Coch Roura and A. Esteves (2008). Adaptive comfort and climate-sensitive architecture: how occupants feel in their homes? 25TH PLEA INTERNATIONAL CONFERENCE, University College Dublin.	Argentina		housung conditions, air temperature, occupant comfort (subjective measure)			Not listed	60				Solutions for thermal comfort in temperate climates.	Thermal comfort
Green, G. and J. Gilbertson (2008). Warm Front Better Health: health impact evaluation of the Warm Front scheme.	UK	Temperature, humidity, fuel, comfort, stress, mental and physical healh.	air temperature, housing conditions, occupant health		Modelling, before and after intervention, what characteristics affect health, warmth.	Not listed	4000 surveys 'half' had data loggers temp and humid	4,000	2000	2 weeks	Evaluation of warm front scheme.	Public Health
Grey, C. N. B., S. Jiang, C. Nascimento, S. E. Rodgers, R. Johnson, R. A. Lyons and W. Poortinga (2017). The short-term health and psychosocial impacts of domestic energy efficiency investments in low-income areas: a controlled before and after study. BMC Public Health 17(1): 1-10.	UK	Thermal satisfaction, 5 point scale.	housung conditions, occupant comfort (subjective measure), socioeconomic status, occupant health (subjective measure)		Questionnaire, 5 point scale, statistical analysis, experimental. Mixed-method Exploratory ((qualitative & quantitative) Analysis: Statistical (x2 test and one way analysis of variance, stepwise & logistic regression, Bivariate analysis).		782 (364 intervention, 418 control)	782			Investing in energy efficiency in low-income communities does not lead to self-reported health improvements in the short term. However, investments increased subjective wellbeing and were linked to a number of psychosocial intermediaries that are conducive to better health.	Public Health
Grey, C. N. B., T. Schmieder-Gaite, S. Jiang, C. Nascimento and W. Poortinga (2017). Cold homes, fuel poverty and energy efficiency improvements: A longitudinal focus group approach. Indoor and Built Environment 26(7): 902-913.	UK	Self reporting thermal comfort, health and welbeing.	health of occupants, age of occupants, socioecomimic status, occupant comfort (subjective measure), housing condition		Focus group discussion, 6 meetings, 3 case studies. Longitudinal focus group approach. Analysis: lived experiences of fuel poverty before and after an energy efficiency intervention.		before (28), after (22) in 3 case studies				Improving the energy efficiency of homes at risk of fuel poverty has a profound impact on wellbeing and quality of life, financial stress, thermal comfort, social interactions and indoor space use.	Energy Poverty
Haddad, S., G. Pignatta, R. Paolini, A. Synnefa and M. Santamouris (2019). AU (NSW)IOP Conference Series: Materials Science and Engineering.	Australia (NSW)	IEQ are temperature, humidity, air velocity, mean radiant temperature.	air temperature			Logtag® TRIX-16, Indoor Air Quality Eggs version 2 Model D	106		106	8 months	More about heat, only mentions cold in introduction, didn't monitor winter.	Energy Poverty
Hagejärd, S., G. Dokter, U. Rahe and P. Femenías (2021). My apartment is cold! Household perceptions of indoor climate and demand-side management in Sweden. Energy Research and Social Science 73.	EU Sweden	Thermal satisfaction, 5 point scale.	housung conditions, air temperature, occupant comfort (subjective measure)		Diary of thermal sensation and satisfaction.		93	93	33 residential buildings	2 weeks	Satisfaction with district heating.	Thermal comfort
Hamilton, I. G., M. Davies, I. Ridley, T. Oreszczyn, M. Barrett, R. Lowe, S. Hong, P. Wilkinson and Z. Chalabi (2011). The impact of housing energy efficiency improvements on reduced exposure to cold - The 'temperature take back factor'. Building Services Engineering Research and Technology 32(1): 85-98.	UK	Secondary analysis of temperature, and relative humidity data.	air temperature, housing conditions	Sensors living room, bedroom, at least 3 weeks.	Assumptions based, statistical analysis.				1600	3 weeks	Modelling approach presented for quantifying the 'temperature take back factor', which is of relevance to environmental and energy modelling.	Miscellaneous
Harrington, L., L. Aye and R. Fuller (2015). Characterising indoor air temperature and humidity in Australian homes. Air Quality and Climate Change 49(4): 21-29.	Australia (all)	Temperature, electricity use.	air temperature, housing conditions	Sensor.	Time series analysis, how indoor related to outdoor temperature.	OmegaWatt			273	6 months	About internal v external temps, no discussion of cold homes.	Miscellaneous
Hayashi, Y., S. M. Schmidt, A. M. Fänge, T. Hoshi and T. Ikaga (2017). Lower physical performance in colder seasons and colder houses: Evidence from a field study on older people living in the community. International Journal of Environmental Research and Public Health 14(6).	Japan	Temperature, survey.	occupant age, socioeconomic status, housing conditions, occupant health	Sensor.	Association between temperature and health.	RTR-503 (T&D Corporation, Nagano, Japan)	162	162	162	2 weeks	Effect of cold homes on older people.	Public Health
Healy, J. D. and J. Peter Clinch (2002). Fuel poverty, thermal comfort and occupancy: Results of a national household - survey in Ireland. Applied Energy 73(3-4): 329-343.	EU Ireland	Thermal comfort.	economic status, occupant comfort (subjective measurement), occupancy patterns	New national household survey.	Statistical relationship between energy poverty and thermal comfort.		1500				Relationship between fuel poverty and thermal comfort.	Energy Poverty

Hiscock, R., A. Asikainen, J. Tuomisto, M. Jantunen, E. Pärjälä and C. E. Sabel (2017). City scale climate change policies: Do they matter for wellbeing? Preventive Medicine Reports 6: 265-270.	EU Finland	Home satisfaction: heating, insulation, air quality, ventilation, thermal comfort and damp.	housung conditions, occupant comfort (subjective measure), air quality, occupant health (subjective measure)		Multivariable statistical modelpostal (27% reponse rate) and online survey, 5 point Likert scale.		680 postal, 102 online	782			Residents who indicated their homes were too cold in winter had significantly lower wellbeing.	Public Health
Howden-Chapman P, Viggers H, Chapman R, O'Dea D, Free S, O'Sullivan K. Warm homes: drivers of the demand for heating in the residential sector in New Zealand. Energy policy. 2009 Sep 1;37(9):3387-99.	New Zealand	Temperature, energy affordability.	air temperature, economic status		Interviews (energy affordability, preferemnce to save money or be warmer.	i_button (Isecondary?)					Many people would prefer to have a little more money than be a little warmer (financial priority/stress).	Energy Poverty
Howden-Chapman, P., H. Viggers, R. Chapman, K. O'Sullivan, L. Telfar Barnard and B. Lloyd (2012). Tackling cold housing and fuel poverty in New Zealand: A review of policies, research, and health impacts. Energy Policy 49: 134-142.	New Zealand	Air temperature, relative humidity.	air temperature, housing conditions, occupant health		Community trial experiment, Data monitored before and after retrofitting.		4407		1350		Resulted in policy programs designed to retrofit insulation and efficient heating into existing houses.	Energy Poverty
Hughes, C. and S. Natarajan (2019). 'The Older I Get, the Colder I Get'—Older People's Perspectives on Coping in Cold Homes. Journal of Housing for the Elderly 33(4): 337-357.	UK	Air temperature, relative humidity, heating practices, thermal comfort.	air temperature, housing conditions, occupant health, occupant age, occupant comfort (subjective measure)		Indoor temps, 4 phases, mixed methods qualitative and quantitative.		43 (aged >65)				Older people do struggle in cold homes and in certain instances have extreme methods of ensuring they can achieve a bearable temperature. Given the aging population, there is likely to be an increasing number of older people needing support in ensuring they can achieve a fair deal for fuel and access necessary benefits.	Energy Poverty
Hughes, C. E. and S. Natarajan (2018). PLEA 2018 - Smart and Healthy within the Two-Degree Limit: Proceedings of the 34th International Conference on Passive and Low Energy Architecture.	China	Temperature, thermal comfort.	housung conditions, air temperature, occupant comfort (subjective measure), occupant age	No full text.	How is thermal comfort related to temperature for older people?						Thermal comfort of older people	Thermal comfort
Hutchinson, E. J., P. Wilkinson, S. H. Hong and T. Oreszczyn (2006). Can we improve the identification of cold homes for targeted home energy-efficiency improvements? Applied Energy 83(11): 1198-1209.	UK	Air temperature, relative humidity, estimated energy efficiency and heating cost.	air temperature, housing conditions, occupant health, occupant age, occupant comfort (subjective measure), occcupancy patterns, socioeconomic status		Sensors, computer-assisted personal interviwew, physical survey, Mixed-methods but dominantly Quantitative Analysis: Statistical Tabulation, logistic regression, multi-variable prediction model.	Gemini TinyTag	463		463	2-4 weeks	Property and household characteristics provide only limited potential for identifying dwellings where winter indoor temperatures are likely to be low, presumably because of the multiple influences on home heating, including personal choice and behaviour.	Energy Poverty
Isaacs, N., K. Saville-Smith, M. Camilleri and L. Burrough (2010). Energy in New Zealand houses: comfort, physics and consumption. Building Research & Information 38(5): 470-480.	New Zealand	Temperature, electricity use.	air temperature, housing conditions, occupant comfort (subjective measure)	Household Energy End-use Study (HEEP).	Statistically analysed energy use v perception of temperature and actual temperature.	Not listed			400		Energy use and temperature.	Miscellaneous
Jesica Fernández-Agüera, Samuel Domínguez-Amarillo, Carmen Alonso, Fernando Martín-Consuegra, Thermal comfort and indoor air quality in low-income housing in Spain: The influence of airtightness and occupant behaviour, Energy and Buildings, 199, 2019, 102-114, ISSN 0378-7788, https://doi.org/10.1016/j.enbuild.2019.06.052.	EU Spain	Air temperature, relative humidity, CO2.	air temperature, housing condition, economic status	Sensors in 6 bnuildings, characterisation of the features of a much larger number of buildings.	Comparison of inddor conditions to outdoors, also bsed on occupancy times, ventillation behavoiur.	Wöhler CDL 210 data logger			6	1 year	Air quality, air tightness.	Thermal Comfort
Johnson, V., J. Totty and D. Sullivan (2013). Improving the energy efficiency of homes in Moreland: warm home cool home and concession assist social research final report.	Australia (Melbourne)	Draughts, codensation, damp, mould,	housung conditions, air temperature, occupant health	Bedford thermal comfort scale (see Griffiths & Boyce 1971; Green & Gilbertson 2008; Wong & Khoo 2003).	Assessing a program to improve thermal comfort and health threough energy efficiency.				56		Factors affecting cold homes, impact on health.	Thermal comfort
Karjalainen S. Thermal comfort and use of thermostats in Finnish homes and offices. Building and Environment. 2009 Jun 1;44(6):1237-45.	EU) Finland	Thermal comfort.	housung conditions, air temperature, occupant comfort (subjective measure)	Questionaire interview survey.							People feel more able to control thermal comfort in homes compared with offices.	Thermal comfort
Karjalainen, S. (2009). Thermal comfort and use of thermostats in Finnish homes and offices. Building and Environment 44(6): 1237-1245.	EU) Finland	Thermal comfort perception and use of thermostats.	housung conditions, occupant comfort (subjective measure), air temperature	Telephone interview survey.	Statistics to determine significant difference between home and office.		3094	3,094			People don't feel as thermally confortable in offices versus homes.	Thermal comfort
Karlen, Carolina Ganem. *Rehabilitación ambiental de la envolvente de viviendas. El caso de Mendoza*. Diss. Universitat Politècnica de Catalunya (UPC), 2006.	Argentina	Temperature.	air temperature, housing conditions	Sensors.	Sensors, different housing types.	Not listed (full text not available)					Energy conservation is a challenge in keeping temperate climate homes warm.	Thermal comfort

Kotol, M., C. Rode, G. Clausen and T. R. Nielsen (2014). Indoor environment in bedrooms in 79 Greenlandic households. Building and Environment 81: 29-36.	Greenland	Temperature, relative humidity, fungi.	air temperature, air quality	Sensor.	Wilcoxon rank sum test, Kruskal–Wallis test). In cases where datasets passed or were close to pass the normality test we also used parametric tests (ANOVA, regression trees).	Temperature/RH/2 External channels data logger (Onset Computer Corp., HOBO® U12-013.				80	Keeping homes warm often compromises ventillation.	Public Health
Lacroix, E. and C. Chaton (2015). Fuel poverty as a major determinant of perceived health: The case of France. Public Health 129(5): 517-524.	EU France	Perception of thermal discomfort (self-reported feeling cold).	occupant comfort (subjective measure), occupant health (subjective measure)	Secondary data : The French database of the Healthcare and Insurance Survey (ESPS) data.	Statistical (Descriptive, Clustering method, Dichotomous probit model).		c22000	c22000	8000		It may be appropriate to reduce the impacts of fuel poverty to provide support for the most vulnerable categories of individuals with respect to the health impacts of fuel poverty and cold homes, e.g., chronic patients who experience difficulty heating their homes.	Public Health
Legendre, B. and O. Ricci (2015). Measuring fuel poverty in France: Which households are the most fuel vulnerable? Energy Economics 49: 620-628.	EU France	Fuel poverty.	socioeconomic status, housing condition, age of occupants	Calculated data based on mathematical equation, French housing survey "Enquête Logement 2006" conducted by the French National Institute of Statistics and Economic Studies .	Fuel poverty = required domestic fuel costs/income >10%, also cited a different metric.		70,000 (60,000 France, 10,000 overseas departments)				Identifying who is fuel poor.	Energy Poverty
Li, B., W. Yu, M. Liu and N. Li (2011). Climatic strategies of indoor thermal environment for residential buildings in Yangtze River Region, China. Indoor and Built Environment 20(1): 101-111.	China	Temperature,. thermal sensation votes.	housung conditions, air temperature, occupant comfort (subjective measure)	Sensor, ASHRAE 55-2004 7-point thermal sensation scale.	Questionnaire data on comfort compared with temperatures.	psychrometer (DHM2, Tianjin Meteorological Instrument Works)		200	200	1 year	Determining comfort paramaters for air conditioning.	Thermal comfort
Limbachiya, V., K. Vadodaria, D. L. Loveday and V. Haines (2012). Identifying a suitable method for studying thermal comfort in people's homes.	UK	Temperature, thermal comfort.	air temperature, occupant comfort (subjective measure)	Fanger's PMV-PPD method (predicted mean vote and percentage people dissatisfied).		hobo			16	3 months	Thermal comfort method comparison.	Thermal comfort
Lloyd, E. L., C. McCormack, M. McKeever and M. Syme (2008). The effect of improving the thermal quality of cold housing on blood pressure and general health: A research note. Journal of Epidemiology and Community Health 62(9): 793-797.	UK	Self-reporting on penetrating damp, growth of mould of varying degrees.	housung conditions, occupant comfort (subjective measure), occupant health	Interview	Experimental, before & after study. Analysis: Statistical (paired t-test).		75 intervention, 40 control				Improving the thermal quality of housing to eliminate damp and mould and produce a comfortable temperature throughout the house has a major impact on the health of the residents. There are also financial benefits for the residents, and indirectly for the NHS.	Public Health
Magalhães, S. M. C., V. M. S. Leal and I. M. Horta (2016). Predicting and characterizing indoor temperatures in residential buildings: Results from a monitoring campaign in Northern Portugal. Energy and Buildings 119: 293-308.	EU Portugal	Indoor temperature On average, households heated their bedrooms and living rooms preferably in December.<br>Bedrooms were usually heated before sleeping hours and living rooms in the evening, when the room is mostly occupied. Thermal comfort 21 °C in the living rooms and 18 °C in the other occupied rooms to achieve an adequate standard of warmth UK Department of	air temperature, housing conditions, occupancy patterns	Measured during winter half-hourly.	Environmental Study<br>Analysis: Statistical(enhanced linear regression with panel-corrected standard errors), Data visualisation.				141		Indoor temperatures are significantly below the comfort levels generally accepted. Results also reinforce the idea that 'cold homes' during winter season are a reality even in the southern European countries.	Miscellaneous
Marí-Dell'Olmo, M., A. M. Novoa, L. Camprubí, A. Peralta, H. Vásquez-Vera, J. Bosch, J. Amat, F. Díaz, L. Palència, R. Mehdipanah, M. Rodríguez-Sanz, D. Malmusi and C. Borrell (2017). Housing Policies and Health Inequalities. International Journal of Health Services 47(2): 207-232.	EU Spain		air temperature, housing conditions, occupant health, occupant comfort (subjective measure), occcupancy patterns, socioeconomic status	Online questionnaire.	Quantitative<br>Analysis: Statistical, Data visualisation.		n=175 adults living in a substandard housing and/or overcrowded and assisted by Ca´ ritas's Direct Assistance Service (DAS);				Possible positive effects of facade insulation interventions on cold-related mortality in women living in social housing; but not in men. Policies on housing energy efficiency can reduce the health consequences of fuel poverty, but need to be free to users, target the most vulnerable groups and be adaptable to their needs.	Energy Poverty
Matsumoto, M., T. Ikaga, Y. Yamakawa, Y. Uchida, S. Murakami, S. Ando, Y. Mitsukura and Y. Hayashi (2018). Relationship between indoor temperature in winter and Brain Healthcare Quotient. 15th Conference of the International Society of Indoor Air Quality and Climate, INDOOR AIR 2018.	Japan	Temperature, survey.	air temperature, occupant health	Sensor, questionnaire.	Compared temp to brain health.		59	59	59		Cold homes impact brain function.	Public Health
Milne, G. and B. Boardman (2000). Making cold homes warmer: The effect of energy efficiency improvements in low-income homes. Energy Policy 28(6-7): 411-424.	UK	Secondary analysis of air temperature, self reported fuel cost. Energy efficiency.	socioeconomic status, housing condition, air temperature	Sensor, survey.	Experiment, (before-after), Mixed-methods.		16		16		The present low levels of warmth and slow rate of improvement in average temperatures will mean that investment in the energy efficiency of the UK housing stock will fail to achieve the predicted energy savings for at least another 15 years.	Energy Poverty
Mu, Zhe, et al. Synergistic effects of temperature and humidity on the symptoms of COPD patients. International journal of biometeorology 61.11 (2017): 1919-1925.	China		air temperature, occupant health	Temperature diary.	General linear mixed model (GLMM) was built for the multilevel ordinal five-level COPD symptom outcome.	standard thermo-hygrometer (WeiHeng, HCT-1)	82			18 months	Temperature and humidity effects on COPD, low temp, high humidity is a risk factor, need 18.2 degrees minimum.	Public Health
Naicker N, Teare J, Balakrishna Y, Wright CY, Mathee A. Indoor temperatures in low cost housing in Johannesburg, South Africa. International journal of environmental research and public health. 2017 Nov;14(11):1410.	S Africa	Apparent temperature (temperature and relative humidity).	air temperature, socioeconomic status		Sensors, interviews.	LogTag, Haxo-8 indoor temperature and RH			59		Variation is greatest in poorer communities. Coping strategies are limited in low cost housing which is poorly constructed.	Energy Poverty

Nakajima, Y., S. M. Schmidt, A. M. Fänge, M. Ono and T. Ikaga (2019). Relationship between perceived indoor temperature and self-reported risk for frailty among community-dwelling older people. International Journal of Environmental Research and Public Health 16(4).	Japan	Self-reporting on perceived temperature (Cold or Warm), economic satisfaction (Unsatisfied or Satisfied).	air temperature, socioeconomic status, age of occupants	Survey.	Cross-sectional study, Quantitative Analysis: Statistical (ANCOVA, MANCOVA).		342, >65s	342			Among those who reported cold homes, only those who were not satisfied with their economic situation reported being at increased risk for frailty. This highlights the potential importance of preventing fuel poverty to prevent frailty.	Energy Poverty
O'Sullivan, K. C., P. Howden-Chapman, D. Sim, J. Stanley, R. L. Rowan, I. K. Harris Clark and L. L. A. Morrison (2017). Cool? Young people investigate living in cold housing and fuel poverty. A mixed methods action research study. SSM - Population Health 3: 66-74.	New Zealand	Perception of cold.	Age (young people), economic status, occupant comfort (subjective measurement)	Online survey, e-interview.	Mixed methods, participatory action research carried out with youth researchers involved at all stages through questionnaire design, analysis, qualitative design, e-interviewing and dissemination of results. Analysis: Descriptive statistics.		656 teens	656			Cold housing and risk of fuel poverty are important problems for young people in New Zealand. Results contribute to the evidence-base for policy targeting of schemes such as the Government-sponsored retrofitting of insulation to households with dependent children	Energy Poverty
Okushima, S. (2016). Measuring energy poverty in Japan, 2004–2013. Energy policy 98: 557-564.	Japan	Energy poverty based on convoluted equations of household expenditure.	socioeconomic status	2004 National Survey of Family Income and Expenditure.	Modelling.		50,000				Determining the extent of energy poverty.	Energy Poverty
Oliveira, A., A. J. R. Cabral, M. Fraga O. Martins, P. Cabral, J. M. Mendes and M. Carreira (2017). Poor housing and stroke mortality in population over 64 years old at the parish level, in mainland Portugal. GeoJournal 82(4): 665-682.	EU Portugal	Self-reporting on the presence of central heating or any form of heating.	housung conditions, occupant comfort (subjective measure), occupant health, occupant age	Equipment: Secondary data from Census. Method: Adopting the parish as the statistical unit. Parishes are Local Administrative Units.	Quantitative analysis: Statistical (ANOVA), GIS (Univariate and multivariate spatial cluster analysis).		4050				Cold homes deserve more attention in stroke prevention and mitigation amongst elderly persons, especially in northwestern continental Portugal.	Public Health
Ormandy, D. (2009). Housing and health in Europe: The WHO LARES project.	EU (6 countries)	Temperature, heating, insullation.	air temperature, occupant health, housing conditions	In-person surveys.			8519		3373			Public Health
Ormandy, D. and V. Ezratty (2012). Health and thermal comfort: From WHO guidance to housing strategies. Energy Policy 49: 116-121.	Global (review)	WHO view of thermal comfort, which is driven by protecting health from both high and low indoor temperatures.	air temperature, occupant comfort (subjective measure)	Secondary analysis: housing surveys has been to use the perception of thermal comfort. Measuring air-temperature but largely considered as impractical.	Literature review.		na				Evidential basis for the WHO guidance. Examines different methods for measuring thermal comfort.	Miscellaneous
Ormandy, D. and V. Ezratty (2016). Thermal discomfort and health: protecting the susceptible from excess cold and excess heat in housing. Advances in Building Energy Research 10(1): 84-98.	EU France, UK	Temperature, thermal comfort.	air temperature, occupant comfort (subjective measure), occupant health	Review	Literature review.						Factors influencing temperature suceptibility.	Thermal comfort
OrtizBeviá MJ, Sánchez-López G, Alvarez-Garcia FJ, RuizdeElvira A. Evolution of heating and cooling degree-days in Spain: trends and interannual variability. Global and Planetary Change. 2012 Jul 1;92:236-47.	EU Spain	Heating degree days.	climate (HDD)		Modelling.						Not expecting significant change in heating degree days in future years	Miscellaneous
Osman, L. M., J. G. Ayres, C. Garden, K. Reglitz, J. Lyon and J. G. Douglas (2008). Home warmth and health status of COPD patients. European journal of public health 18(4): 399-405.	UK	Temperature sensors.	air temperature, occupant health	Sensor	Temperature data compared with respiratory health.	Escort iLOG™ datalogger	148		148	1 week	21 degrees for 9 hrs a day is assoc with better health for COPD patients.	Public Health
Papada, L. and D. Kaliampakos (2016). Measuring energy poverty in Greece. Energy Policy 94: 157-165.	EU Greece	Energy usage, perception of housing conditions, inability to keep warm.	housing conditions, occupant comfort (subjective measurement)	Telephone interview.	Statistical analysis of contributing factors.				400		Strategies to combat energy poverty.	Energy Poverty
Peralta, A., L. Camprubí, M. Rodríguez-Sanz, X. Basagaña, C. Borrell and M. Marí-Dell'Olmo (2017). Impact of energy efficiency interventions in public housing buildings on cold-related mortality: A case-crossover analysis. International Journal of Epidemiology 46(4): 1192-1201.	EU Spain	Outdoor meteorological data.	housung conditions, occupant comfort (subjective measure), occupant health, occupant age	Equipment: Secondary data Methods: Temperature lag windows covering the day of the death and the previous 20 days	Time-stratified case-crossover. Analysis: Statistical. Met data correlated with mortality data.		na				Energy efficiency facade retrofitting (EEFR) interventions had differentiated effects on cold-related mortality in men and women. Differentiated effects were also observed by cause, educational level and age.	Public Health
Petrova, S., M. Gentile, I. H. Mäkinen and S. Bouzarovski (2013). Perceptions of thermal comfort and housing quality: exploring the microgeographies of energy poverty in Stakhanov, Ukraine. Environment and Planning A 45(5): 1240-1257.	Ukraine	Thermal comfort perception.	occupant comfort (subjective measure), housing conditions, socioeconomic status	Stakhanov Health Interview Survey.	Statistical analysis by building type etc.		3000	3,000			Identifying households with inadequate energy.	Thermal comfort

Pierse, Nevil, et al. Modelling the effects of low indoor temperatures on the lung function of children with asthma. J Epidemiol Community Health 67.11 (2013): 918-925.	New Zealand	Temperature.	air temperature, occupant health, occupant age	Sensor	Correlation with lung function.	Temperature meters	409		409	128 days	Indoor temperature related to lung function.	Public Health
Pollard, A. R. (2018). Could Damp Homes be Too Cold/underheated?, BRANZ.	New Zealand	Temperature, relative humidity.	air temperature, housing conditions	Used existing studies, incl BRANZ Household Energy End-use Project (HEEP).		Electronic temperature logger			400		Could damp be exacerbated by cold?	Public Health
Pollard, A., T. Jones, S. Sherratt and R. A. Sharpe (2019). Use of simple telemetry to reduce the health impacts of fuel poverty and living in cold homes. International Journal of Environmental Research and Public Health 16(16).	UK	Temperature, self-reporting?	air temperature, socioeconomic status	Thermometer, questionnaire.			34 thermometer, 22 survey		34			Public Health
Pullinger, M., N. Berliner, N. Goddard and D. Shipworth (2022). Domestic heating behaviour and room temperatures: Empirical evidence from Scottish homes. Energy and Buildings 254: 111509.	UK	Room temperature, heating use.	air temperature, housing conditions	IDEAL Household Energy Dataset.	Statistical analysis to look at usage and temp by day of week/time/ household characteristics etc.	Wall-mounted sensors reported wirelessly.			255	55-673 days	Actual home temps are lowere than standard proceedure assumptions.	Miscellaneous
Reyes, R., A. Schueftan, C. Ruiz and A. D. González (2019). Controlling air pollution in a context of high energy poverty levels in southern Chile: Clean air but colder houses? Energy Policy 124: 301-311.	Chile	Energy consumption, thermal comfort (defines thermal comfort as 21 degrees or more in living room.	air quality, air temperature, socioeconomic status, housing condition	Sensor.	Energy poverty using 10% threshold, time temp below threshold (21 degrees) was 65%.	Speck® sensors	300 surveys, 80 homes monitored temp	300	80	<1 month	Fuel poverty is very prevalant and is related to air pollution, particularly wood burning	Energy Poverty
Riggs, L., M. Keall, P. Howden-Chapman and M. G. Baker (2021). Environmental burden of disease from unsafe and substandard housing, New Zealand, 2010–2017. Bulletin of the World Health Organization 99(4): 259-270.	New Zealand	Perception of cold homes, dampness, mould.	housung conditions, occupant comfort (subjective measure), occupant health	Secondary data from National survey.	Quantitative analysis: Cost-analysis.		8795	8,795			Damp and mouldy housing accounted for a substantial proportion of the burden of disease in New Zealand. Improving people's living conditions could substantially reduce total hospitalization costs and potentially improve quality of life.	Public Health
Rudge, J. and R. Gilchrist (2005). Excess winter morbidity among older people at risk of cold homes: A population-based study in a London borough. Journal of Public Health 27(4): 353-358.	UK	Indirect measure i.e extent of homes with energy efficiency ratings, lack of central heating, SAP rating of dwellings.	housung conditions, occupant health, occupant age	Hospital Episode Statistics, population study.	Observational, population-based study.		460				Supporting evidence of a relationship between energy inefficient housing and winter respiratory disease among older people, with public health implications for increasing health-driven energy efficiency housing interventions.	Public Health
Rudge, J. and R. Gilchrist (2007). Measuring the health impact of temperatures in dwellings: Investigating excess winter morbidity and cold homes in the London Borough of Newham. Energy and Buildings 39(7): 847-858.	UK	Secondary indirect data on energy efficiency of dwellings.	housung conditions, occupant health	Hospital Episode Statistics, population study.	Statistical modelling (Quantitative).		460				A new methodology is developed that links the risk of cold homes with excess winter hospital episodes, demonstrating its potential for identifying small areas for priority action on improving domestic energy efficiency in terms of health as well as the environment.	Public Health
Rupp, R. F., N. G. Vásquez and R. Lamberts (2015). A review of human thermal comfort in the built environment. Energy and buildings 105: 178-205.	Global (review)	Thermal comfort perception.	occupant comfort (subjective measure), housing conditions	Literature review.	Determining standards for thermal comfort across literature.						Effect of built environment on thermal comfort.	Thermal comfort
S. Bouzarovski, S. Petrova<br>A global perspective on domestic energy deprivation: overcoming the energy poverty–fuel poverty binary<br>Energy Res. Soc. Sci., 10 (2015), pp. 31-40, 10.1016/J.ERSS.2015.06.007	Global	Thermal comfort 17 degrees at night, 21 during day.	housing condition, economic status, occupant comfort (subjective measurement)								Overcoming energy poverty.	Energy Poverty
Saeki, K., K. Obayashi and N. Kurumatani (2016). Indoor cold exposure and nocturia: A cross-sectional analysis of the HEIJO-KYO study. BJU International 117(5): 829-835.	Japan	Air temperature.	air temperature, occupant health	Sensor	Cross-sectional Analysis Approach. Analysis: Statistical (univariate association linear regression, ANOVA etc.).	Thermochron iButton DS1922L				48 hrs	Indoor cold exposure during the daytime was independently associated with nocturia among elderly participants. The explanation for this association may be cold-induced detrusor overactivity. The prevalence of nocturia could be reduced by modification of the indoor thermal environment.	Public Health
Saeki, K., K. Obayashi and N. Kurumatani (2017). Platelet count and indoor cold exposure among elderly people: A cross-sectional analysis of the HEIJO-KYO study. Journal of Epidemiology 27(12): 562-567.	Japan	Air temperature.	air temperature, occupant health, occupant age	Sensor	Cross-sectional Analysis Approach<br>Analysis: Statistical (univariate association linear regression, ANOVA etc.).	Thermochron iButton DS1922L				48 hrs	Significant and independent association between lower indoor temperature and higher PLT count among elderly in winter.	Public Health

Saeki, K., K. Obayashi, J. Iwamoto, N. Tone, N. Okamoto, K. Tomioka and N. Kurumatani (2014). Stronger association of indoor temperature than outdoor temperature with blood pressure in colder months. Journal of hypertension 32(8): 1582-1589.	Japan	Indoor temperature.	air temperature, occupant health	Sensor	Matched temperature to blood pressure.	Fixed thermosensor	868		868	48 hrs	Reducing winter mortality.	Public Health
San Miguel-Bellod, J., P. González-Martínez and A. Sánchez-Ostiz (2018). The relationship between poverty and indoor temperatures in winter: Determinants of cold homes in social housing contexts from the 40s–80s in Northern Spain. Energy and Buildings 173: 428-442.	EU Spain	Air-temperature & relative humidity data. Self-reporting on building and heating system characteristics, fuel cost.	air temperature, socioeconomic status, housing condition, occupant comfort (subjective measure)	i) Sensors (portable) ii) face-to-face structured interview iii)field survey.	Mixed-methods but mostly Quantitative<br>Analysis: Mediation analysis.		112		112		Framework for comparing the assumptions derived from other contexts to the situation in Spain about the effects of space heating and housing deprivation on thermal conditions, provides a basis for reviewing some possible intervention, and aims to raise social and political awareness further about this issue.	Energy Poverty
Sánchez-Guevara Sánchez, C., F. J. Neila González and A. Hernández Aja (2018). Energy poverty methodology based on minimal thermal habitability conditions for low income housing in Spain. Energy and Buildings 169: 127-140.	EU Spain	Simulated data (Dwelling thermal performance).	socioeconomic status, housing condition, air temperature	Equipment: Simulation software Methods: input data collected & then simulated.	Simulation study & Case-study Method<br>Analysis: Data visualization.		27 (3 blocks of 9 units)				Method for evaluating energy poverty in low income dwellings The resulting method constitutes a useful tool for the identification of households suffering from energy poverty as well as the degree of the need they require (Fig. 1). Finally, the method poses an aid in the decision-making processes related to dwelling energy retrofitting actions and policy.	Energy Poverty
Santamouris, M., S. Alevizos, L. Aslanoglou, D. Mantzios, P. Milonas, I. Sarelli, S. Karatasou, K. Cartalis and J. Paravantis (2014). Freezing the poor—Indoor environmental quality in low and very low income households during the winter period in Athens. Energy and Buildings 70: 61-70.	EU Greece	Indoor temperature, energy use.	air temperature, socioeconomic status	Sensor	Comparison to ambient temperatures.	Miniature temperature sensor.			43	4 months	The poor can't afford to heat their homes.	Public Health
Sartini, C., P. Tammes, A. D. Hay, I. Preston, D. Lasserson, P. H. Whincup, S. G. Wannamethee and R. W. Morris (2018). Can we identify older people most vulnerable to living in cold homes during winter? Annals of Epidemiology 28(1): 1-7.e3.	UK	Self-reported feeling cold.	air temperature, socioeconomic status, occupant health	Survey	Logistic regression.		1402				Cold homes increase mortality risk.	Public Health
Sherriff G, Moore T, Berry S, Ambrose A, Goodchild B, Maye-Banbury A. Coping with extremes, creating comfort: User experiences of 'low-energy'homes in Australia. Energy Research & Social Science. 2019 May 1;51:44-54.	Australia (SA)	Experiences.	housing conditions, occupant comfort (subjective measurement)								Low energy homes improve comfort, but don't eliminate the problem.	Energy Poverty
Shipworth, M. (2011). Thermostat settings in English houses: No evidence of change between 1984 and 2007. Building and Environment 46(3): 635-642.	UK	Thermostat settings.	air temperature, housing conditions	INT84: Intensive 1984 home energy use survey, CARB07: Carbon reduction in buildings 2007 home energy use survey	Compared two surveys to discern increase in thermostat settings.		149				Are people heating homes to higher temp? (no), why then are we using more energy? Area and duration heated may have increased.	Miscellaneous
Shiue, I. (2016). Cold homes are associated with poor biomarkers and less blood pressure check-up: English Longitudinal Study of Ageing, 2012–2013. Environmental Science and Pollution Research 23(7): 7055-7059.	UK	Temperature.	air temperature, occupant age, occupant health	Sensors.	Longitudinal Study, Mixed-methods<br>Analysis: Statistical (t test or chi-square test), Software STATA version 13.0		18 (temp recording), 7997 onler adults		18		Age was inversely associated with people who resided in cold homes or who tended not to have blood pressure check-up. Those who resided in cold homes had higher blood pressure readings, worse handgrip, lower vitamin D levels, higher cholesterol levels, higher insulin-like growth factor levels, higher haemoglobin levels, lower level of white blood cell count	Public Health
Simoes SG, Gregório V, Seixas J. Mapping fuel poverty in Portugal. Energy Procedia. 2016 Dec 1;106:155-65.	EU Portugal	Fuel poverty.	socioeconomic status, housing condition, air temperature, occupant age, climate (HDD)		Modelled fuel poverty.						20% of households experience fuel poverty	Energy Poverty
Singh, M. K., S. Attia, S. Mahapatra and J. Teller (2016). Assessment of thermal comfort in existing pre-1945 residential building stock. Energy 98: 122-134.	EU Belgium	Temperature, thermal comfort.	air temperature, occupant comfort (subjective measure), housing conditions	Sensor, survey.	Analysed by building characteristics and occupant behaviours.	HOBO-U12 RH			20	6 months	Thermal performance of housing.	Thermal comfort
Song, C., L. Huang, Y. Liu, Y. Dong, X. Zhou and J. Liu (2020). Effects of indoor thermal exposure on human dynamic thermal adaptation process. Building and Environment 179.	China	Comfort voting.<br>Thermal record in climate chamber.	air temperature, occupant comfort (subjective measure)	i) Questionnaire, ii)Climate chamber (Thermo recorder, Electrocardiograph, Globe thermometer)	Experimental Thermal Comfort Study<br>Analysis: Data visualization, statistical		30	30	30		Implications for the control of indoor thermal environments based on human adaptability.	Thermal comfort
Stafford, B. (2015). The social cost of cold homes in an English city: Developing a transferable policy tool. Journal of Public Health (United Kingdom) 37(2): 251-257.	UK	Estimated social cost of cold homes.	socioeconomic status, occupant health	Secondary data collected from i) Sheffield Local Authority ii) HHSRS •Methods: Estimated	Quantitative Study<br>Analysis: cost–benefit analysis						Estimate of the monetised 1 year social cost of cold homes in the City of Sheffield.	Public Health

Stewart, J. and S. Dhesi (2016). Affordable warmth: Housing strategies for older people. Housing, Care and Support 19(1): 23-31.	UK	Affordable warmth interventions.	occupant age, socioeconomic status	Semi-structured interviews.	Qualitative- Review-based Anaysis: Desk-top analysis, software Atlas		50				Consolidated policy, research, evidence and good practice around strategies tackling fuel poverty and affordable warmth for older people aged over 60 to support the development of more effective services for this life course stage and to tackle physical and mental health inequalities.	Energy Poverty
Sun, X., S. Zhu, H. Zhu, R. Duan and J. Wang (2019). Comparison and analyses of two thermal performance evaluation models for a public building. Open Physics 17(1): 916-926.	China	Indoor temperature, thermal performance.	air temperature, occupant comfort (subjective measure)	Synthetic data modelling.	Simulation Analysis: thermodynamics model with synthetic data				1		Two models based on thermodynamics are given to evaluate building thermal performance.	Thermal comfort
Sunikka-Blank, M. and R. Galvin (2021). Single parents in cold homes in Europe: How intersecting personal and national characteristics drive up the numbers of these vulnerable households. Energy Policy 150.	EU and UK (28 countries)	Heating degree days, arrears on energy bills, poor housing percentage, gas price.	socioeconomic status, housing condition, climate (HDD)	Secondary data base Eurostat2 (2020).	Quantitative case-crossover approach Analysis: Conditional logistic regression		252				Upward pressure on the percentage of single parent households unable to heat their homes.	Energy Poverty
Tammes, P., C. Sartini, I. Preston, A. D. Hay, D. Lasserson and R. W. Morris (2018). Use of primary care data to predict those most vulnerable to cold weather a case-crossover analysis. British Journal of General Practice 68(668): e146-e156.	UK	Indirect and secondary temperature data.	air temperature, occupant health, occupant age	Secondary data from Met office & Clinical Practice Research Datalink.	Quantitative case-crossover approach Analysis: Conditional logistic regression		34777, >65 yrs				Unlikely that GPs can identify older patients at highest risk of cold-related death using routinely available data.	Public Health
Tartarini, F. (2017). Impact of temperature and indoor environmental quality in nursing homes on thermal comfort of occupants and agitation of residents with dementia.	Australia (Woolongong area, NSW)	Temperature, PMV thermal sensation votes.	air temperature, occupant comfort (subjective measure), occupant age, occupant health	Sensor, survey (web based, to staff members only).		iButton® sensors/data loggers			6	up to 5 months	Welfare of people in aged care homes.	Public Health
Tartarini, F., P. Cooper and R. Fleming (2017). Thermal environment and thermal sensations of occupants of nursing homes: a field study. Procedia Engineering 180: 373-382.	Australia (Woolongong area, NSW)	Temperature, PMV thermal sensation votes.	air temperature, occupant comfort (subjective measure), occupant age	Sensor, survey.	Statistical analysis thermal comfort.	iButton® sensors/data loggers	252 surveys	252	6		Assessing indoor environmental quality.	Public Health
Tartarini, F., P. Cooper and R. Fleming (2018). Thermal perceptions, preferences and adaptive behaviours of occupants of nursing homes. Building and Environment 132: 57-69.	Australia (Woolongong area, NSW)	Temperature, PMV thermal sensation votes.	air temperature, occupant comfort (subjective measure), housing conditions	Sensor, survey.	Statistics: compared results for residents and non-residents.	iButton® sensors/data loggers	509 surveys	509		while they were surveyed <1 hr	Estimated comfort range for residents was 19.1–26.2 °C.	Thermal comfort
Thomson, H., S. Bouzarovski and C. Snell (2017). Rethinking the measurement of energy poverty in Europe: A critical analysis of indicators and data. Indoor and Built Environment 26(7): 879-901.	EU	Access,. Affordability, energy efficiency, needs.	socioeconomic status, housing condition, occupant health	Literature review.	Looking at best way to measure energy poverty.						Measuring energy poverty	Energy Poverty
Tod, A. M., A. Lusambili, C. Homer, J. Abbott, J. M. Cooke, A. J. Stocks and K. A. McDaid (2012). Understanding factors influencing vulnerable older people keeping warm and well in winter: A qualitative study using social marketing techniques. BMJ Open 2(4).	UK	Home heating, behaviour and experience (self report).	occupant comfort (subjective measure), occupant age, housing conditions	Telephone interview.	Qualitative study incorporating in-depth, semi-structured individual and group interviews, Analysis: framework analysis and social marketing segmentation techniques.		118, various groupings				How and why vulnerable older people may be at risk of a cold home.	Public Health
Tod, A. M., P. Nelson, A. C. De Chavez, C. Homer, V. Powell-Hoyland and A. Stocks (2016). Understanding influences and decisions of households with children with asthma regarding temperature and humidity in the home in winter: A qualitative study. BMJ Open 6(1).	UK	Self-reporting on cold, damp, safe and healthy temperatures, affordability of heating and fuel payments.	occupant comfort (subjective measure), occupant age (children), housing conditions	Semistructured individual interviews, ii) second, group interviews.	Qualitative study Analysis: a framework analysis approach. 24 27 NVivo V.10 was used to facilitate this.		105 various categories	105			Identifies a range of psychological and contextual influences on parents that may inadvertently place a child with asthma at risk of cold, damp and worsening health in a home. Parents made 'trade-offs' that drove their behaviour regarding the temperature and humidity of the home, including partial self-disconnection from their energy supply.	Public Health
Umishio, W., T. Ikaga, K. Kario, Y. Fujino, M. Suzuki, S. Ando, T. Hoshi, T. Yoshimura, H. Yoshino and S. Murakami (2021). Electrocardiogram abnormalities in residents in cold homes: a cross-sectional analysis of the nationwide Smart Wellness Housing survey in Japan. Environmental Health and Preventive Medicine 26(1).	Japan	Temperature.	air temperature, occupant health	Sensor	Compared in bands: warm, slightly cold, cold, chi-squared test.		1480		1480	2 weeks	ECG abnormalities related to cold homes.	Public Health
Umishio, W., T. Ikaga, Y. Fujino, S. Ando, T. Kubo, Y. Nakajima, T. Hoshi, M. Suzuki, K. Kario, T. Yoshimura, H. Yoshino and S. Murakami (2020). Disparities of indoor temperature in winter: A cross-sectional analysis of the Nationwide Smart Wellness Housing Survey in Japan. Indoor Air 30(6): 1317-1328.	Japan	Temperature, self-reporting on heating device.	air temperature, housing conditions	Sensors, 14 day survey and diary.	Cross-sectional analyses Analysis: Statisical	Sensors(thermo-hygrometer) ii) survey ii) Participants Diary			2190	14 day	indoor temperatures appear lower than in Euro-American countries because of low insulation standards and use of partial intermittent heating.	Miscellaneous

US EPA. (2009) Indoor Air Quality Tools for Schools. https://www.epa.gov/sites/default/files/2014-08/documents/reference_guide.pdf	USA	Temperature, relative humidity.	air temperature, air quality	Guideline							Ventilation, comfort, air conditioning	Public Health
Vadodaria, K. (2014). Thermal comfort in UK Homes: how suitable is the PMV approach as a prediction tool? , Loughborough University.	UK		occupant comfort (subjective measure)	Sensor, online questionnaire.	How well did perception match with temperature.	Hobo U12-013	20			2 hrs	Usefulness of PMV as a measure (said it was good).	Thermal comfort
Van Hoof, J., (2008). Forty years of Fanger's model of thermal comfort: comfort for all?. Indoor Air. 18:3, 182-201	NA, review	PMV, alternative measures of thermal comfort.	occupant comfort (subjective measure)								Move from a predicted mean vote to comfort for all.	Thermal comfort
Van Hoof, J., H. Kort, J. Hensen, M. Duijnstee and P. Rutten (2010). Thermal comfort and the integrated design of homes for older people with dementia. Building and Environment 45(2): 358-370.	UK	Air temperature.	air temperature, occupant health, occupant age	Sensor.	Compared temperature with health outcomes.		148		148	1 week	21 degrees for 9 hrs/day brings better health outcomes for COPD patients.	Public Health
Walker, R., P. McKenzie, C. Liddell and C. Morris (2012). Area-based targeting of fuel poverty in Northern Ireland: An evidenced-based approach. Applied Geography 34: 639-649.	EU N Ireland	Estimated heating burden based on degree days and oil prices.	climate (HDD), economic factors	Calculated data based on mathematical equation.	Using Geographic Information Systems (GIS) techniques, to develop a small area fuel poverty risk index for Northern Ireland using a range of environmental and socio-economic variables, statitsical areas.		Small area (OA) level (averaging 125 households per OA)				Areas at highest risk of fuel poverty.	Energy Poverty
Wallace, L., S. J. Emmerich and C. Howard-Reed (2002). Continuous measurements of air change rates in an occupied house for 1 year: the effect of temperature, wind, fans, and windows. Journal of Exposure Science & Environmental Epidemiology 12(4): 296-306.	USA	Temperature, air change, wind, behaviour.	air temperature, housing conditions	Used existing studies, incl BRANZ Household Energy End-use Project. (HEEP)		Not listed			1	1 year	Air change rates versus temp.	Miscellaneous
Watson, P. and S. Watson (2017). Warm house, Cold house: A review of measures of thermal comfort used in Get Bill Smart's energy efficiency assessments. Energy Procedia.	Australia (Tasmania)	Thermal comfort, energy use.	occupant comfort (subjective measure), housing conditions	Longitudinal interviews, loggers.		Stand-alone USB temperature (DBT) and humidity (RH) loggers			51 (thermal comfort and energy use), 510 interviews	15 months	Developing thermal comfortort and energy indicators.	Thermal comfort
Willand, N. and R. Horne (2018). "They are grinding us into the ground" – The lived experience of energy (in)justice amongst low-income older households. Applied Energy 226: 61-70.	Australia (Melbourne)	Temperature monitoring during the pre- and post-intervention.<br>Self-reporting on heating and paying energy bills, keeping warm and affording energy before and after simple retrofits.	occupant age, socioeconomic status, air temperature, occupant comfort	Sensor, i) householder surveys iii) semi-structured interviews iv) field observations.	Experimental (before-after study) Mixed methods evaluation of a quasi-randomised controlled trial of residential energy efficiency improvement<br>Analysis: The interpretation of the quantitative results in this paper is descriptive and focus on clinical significance.	Sensor (HOBO UX100-3) ii) householder surveys iii) semi-structured interviews iv) field observations	30	30	30		Energy justice by revealing how the principles of energy justice manifest at the domestic scale.	Energy Poverty
Wookey R, Bone A, Carmichael C, Crossley A. Minimum home temperature thresholds for health in winter–a systematic literature review. London: Public Health England. 2014.	UK	Temperature.	air temperature, occupant health		Literature review.						Minimum thresholds.	Public Health
Wright, F. (2004). Old and cold: Older people and policies failing to address fuel poverty. Social Policy and Administration 38(5): 488-503.	UK	Self-reporting: felt warm or cold during the recent winter, heating systems, insulation, winter fuel bills costing, experiences of keeping the home warm in winter.	occupant age, socioeconomic status, occupant comfort (subjective measure), housing conditions	Detailed questionnaire for factual information such as, insulation, income and expenditure on fuel in the preceding winter quarter, and ii) an in-depth tape-recorded exploration of views and experiences of keeping the home warm in winter.			64	64			Part of many older people's culture is to turn off heating during part of the day in winter. A cold bedroom with an open window throughout the night remains a common practice.	Miscellaneous
Wright, M. K., D. M. Hondula, P. M. Chakalian, L. C. Kurtz, L. Watkins, C. J. Gronlund, L. Larsen, E. Mallen and S. L. Harlan (2020). Social and behavioral determinants of indoor temperatures in air-conditioned homes. Building and Environment 183.	USA	Monitored data: temperature & relative humidity.<br>Self-reporting on thermostat thermostat setting, heat related illness.	socioeconomic status, air temperature, occupant health	Sensor, survey.	Quantitative, statistical.	HOBO UX100-011	46		46	1 month	Households may be sacrificing other necessities to keep their homes comfortable.	Energy Poverty
Xu, C., S. Li, X. Zhang and S. Shao (2018). Thermal comfort and thermal adaptive behaviours in traditional dwellings: A case study in Nanjing, China. Building and Environment 142: 153-170.	China	Thermal comfort and thermal adaptive behaviours.	occupant comfort (subjective measure), housing conditions	survey, sensor	Mainly descriptive statistics.	TH21E Digital temperature and humidity meter	234	234	234	while they were surveyed <1 hr	thermal comfortort and adaptive behaviours.	Thermal comfort

You, Y. and S. Kim (2019). Who lives in and owns cold homes? A case study of fuel poverty in Seoul, South Korea. Energy Research and Social Science 47: 202-214.	S Korea	Thermal efficiency of dwellings in terms of heating energy consumption (LNG gas consumption data per building).<br>Heat loss (measured the heterogeneous insulation and heat bridges).	home condition, socioeconomic status	Survey, infrared thermography (heat loss).	Quantitative, statistical, ANOVA.		360				Fuel poverty problems occur in different ways according to the social and spatial contexts and thus need a more contextualised policy approach beyond the simplistic criteria of household income and heating cost.	Energy Poverty
Yu, W., B. Li, R. Yao, D. Wang and K. Li (2017). A study of thermal comfort in residential buildings on the Tibetan Plateau, China. Building and Environment 119: 71-86.	China	Temperature, thermal comfort.	air temperature, occupant comfort (subjective measure), housing conditions	survey, sensor	Correlation between temperature and other (eg. Clothing), a PMV model.	SWEMA Black Ball temperature instrument.	527	527	527	while they were surveyed <1 hr	Modelling thermal comfort.	Thermal comfort
Zheng, W., T. Shao, Y. Lin, Y. Wang, C. Dong and J. Liu (2022). A field study on seasonal adaptive thermal comfort of the elderly in nursing homes in Xi'an, China. Building and Environment 208: 108623.	China	Temperature, thermal comfort.	air temperature, occupant comfort (subjective measure), occupant age	survey, sensor	Correlation between temperature and other (eg. Clothing), a PMV model.	Instruments comply with ISO 7726–2002	213	213	213	while they were surveyed <1 hr	Thermal comfortort of the elderly.	Thermal comfort
